# Divergence at the edges: peripatric isolation in the montane spiny throated reed frog complex

**DOI:** 10.1186/s12862-015-0384-3

**Published:** 2015-07-01

**Authors:** Lucinda P. Lawson, John M. Bates, Michele Menegon, Simon P. Loader

**Affiliations:** Committee on Evolutionary Biology, University of Chicago, 1025 E. 57th St. Culver Hall 402, Chicago, IL 60637 USA; Life Sciences, Field Museum of Natural History, 1400 S. Lake Shore Dr., Chicago, IL 60605 USA; Department of Biological Sciences, University of Cincinnati, 614 Rieveschl Hall, Cincinnati, OH 45220 USA; Tropical Biodiversity Section, Science Museo of Trento, Via della Scienza e del lavoro, 38122 Trento, Italy; Biogeography Research Group, Department of Environmental Sciences, University of Basel, Basel, 4056 Switzerland

**Keywords:** Speciation, Eastern Afromontane Biodiversity Hotspot, Coalescent analysis, Demography

## Abstract

**Background:**

Peripatric speciation and peripheral isolation have uncertain importance in species accumulation, and are largely overshadowed by assumed dominance of allopatric modes of speciation. Understanding the role of different speciation mechanisms within biodiversity hotspots is central to understanding the generation of biological diversity. Here, we use a phylogeographic analysis of the spiny-throated reed frogs and examine sister pairings with unbalanced current distributional ranges for characteristics of peripatric speciation. We further investigate whether forest/grassland mosaic adapted species are more likely created through peripatric speciation due to instability of this habitat type.

**Results:**

We reconstructed a multi-locus molecular phylogeny of spiny-throated reed frogs which we then combined with comparative morphologic data to delimit species and analyze historical demographic change; identifying three new species. Three potential peripatric speciation events were identified along with one case of allopatric speciation. Peripatric speciation is supported through uneven potential and realized distributions and uneven population size estimates based on field collections. An associated climate shift was observed in most potentially peripatric splits. Morphological variation was highest in sexually dimorphic traits such as body size and gular shape, but this variation was not limited to peripatric species pairs as hypothesized. The potentially allopatric species pair showed no niche shifts and equivalent effective population sizes, ruling out peripatry in that speciation event. Two major ecological niche shifts were recovered within this radiation, possibly as adaptations to occupy areas of grassland that became more prevalent in the last 5 million years. Restricted and fluctuating grassland mosaics within forests might promote peripatric speciation in the Eastern Arc Biodiversity Hotspot (EABH).

**Conclusions:**

In our case study, peripatric speciation appears to be an important driver of diversity within the EABH biodiversity hotspot, implying it could be a significant speciation mechanism in highly fragmented ecosystems. Extensive peripatric speciation in this montane archipelago may explain the abundance of discrete lineages within the limited area of the EABH, as inferred in remote island archipelagos. Future phylogenetic studies incorporating demographic and spatial analyses will clarify the role of peripatric speciation in creating biodiversity hotspots.

**Electronic supplementary material:**

The online version of this article (doi:10.1186/s12862-015-0384-3) contains supplementary material, which is available to authorized users.

## Background

Isolation has been historically considered the most frequent mechanism to limit gene flow, promoting divergence of lineages leading to speciation [1, 2]. The two dominant proposed forms of isolation have been vicariant allopatric (dichopatric) and peripatric, while sympatric and parapatric speciation appear less common [3] (see below). The classical model of vicariant allopatry starts with an ancestral distribution, which is split into large, disjointed populations [2]; hereafter referred to as “vicariant allopatry” or simply “allopatry”. Numerous examples have been cited demonstrating the process of allopatry in driving speciation (summarized in [3, 4]), and the signal of vicariant barriers limiting gene flow has been shown in many comparative phylogeographic studies [5–8]. In peripatric speciation, a small population is isolated at the edge of a larger population and rapidly diversifies, often crossing a previous barrier to dispersal and/or occupying a newly available ecological niche/habitat type. Over short periods of evolutionary time, peripatric events leave different evolutionary footprints from allopatry in terms of range, historical demography, and degree of ecological and morphological divergence [9]. Parapatric speciation events also involve a shift in habitat requirements or ecological niche, though unlike in peripatric speciation, there are no physical barriers separating divergent lineages. Sympatric speciation, where species diverge within the same environment without spatial or ecological barriers, has rarely if ever been observed [3]. New analytical approaches using multiple types of data enable testing for these processes in greater detail [10–13].

Different speciation processes produce predictable outcomes in the molecular, morphological, and geographic relationships among lineages. In contrast to the stable population sizes associated with allopatric speciation, a peripatric speciation event yields genetic variation consistent with bottlenecks and low founder population numbers [14]. Peripatric species are also more likely to be ecologically divergent, as they have crossed a barrier to dispersal that previously limited occupation of new habitat at the edge of the ancestral range [3, 15]. Allopatric species, in contrast, more often retain their ecological niche due to the similarity of habitats isolated by vicariant barriers [16–18]. Morphologically, divergence in physical traits, especially those shaped by sexual selection, are predicted to be especially affected in peripatric populations both due to drift in small populations and as reinforcement between sister lineages in close proximity [19–22]. Quantifying molecular, ecological, and morphological metrics among sister lineages can therefore distinguish between these two modes (allopatry and peripatry) in species formation.

Across the African continent, speciation patterns are largely consistent with fragmentation of formally continuous forests through fluctuating climate [23–28]. Such patterns occur in the Eastern Afromontane Biodiversity Hotspot (EABH). Here, isolation of forest endemics on refugial mountain tops appears to be a common driver of exceptional levels of endemism and species diversity (e.g., [26, 29–31]). However, in part due to incomplete phylogenetic and phylogeographic sampling and imprecise distribution models, little attention has been paid to whether regional-scale fragmentation (vicariance/allopatric) or local-scale expansions or niche shifts (peripatric) are the dominant speciation process within this sky-island system.

We evaluate an insular montane radiation of spiny-throated reed frogs within the EABH and assess regional-scale and local-scale speciation mechanisms. Deciphering these two processes for individual radiations is imperative to understand diversification in tropical ecosystems, as both niche conservatism and niche divergence appear to play a major role in amphibian speciation [32, 33]. Establishing the speciation processes involved in the spiny-throated reed frog radiation allows for direct comparisons with other EABH endemic radiations as well as linking these processes to montane and island archipelagos around the world.

Within the EABH, the arrangement of montane areas of varying size and distance from each other provides an ideal setting in which to compare mechanisms of divergence. The mountains of the EABH system are dominated today by two forest vegetation types: rainforest found approximately between 700-1800 m or lower depending upon the mountain block and aspect (referred to as “rainforest” hereafter), and a montane forest/grassland mosaic (referred to as “mosaic” hereafter) above ca. 1800 m in the southern EABH [34]. The extent and distribution of these biomes throughout the EABH has varied through time, with a gradual shift towards increased grasslands from 4 mya onward [35]. The areas of high montane forest/grassland mosaic have historically expanded and contracted and at times likely vanished or become restricted to extremely narrow refugia as forests expanded [36]. Lower elevation rainforest habitats fluctuated to a lesser extent, and were likely persistently stable on montane slopes despite habitat cycling in the adjacent lowland savannahs (classically outlined by [37]; see [38–41]). The larger fluctuations, smaller potential distributions, and the relative newness of mosaic habitat compared to mid-elevation rainforests, may make the mosaic habitat biome more susceptible to peripatry mechanisms than the more persistent rainforest biome. Though other biome-specific patterns of diversification are known [42, 43], whether peripatric speciation could also be biome-specific is less well documented [44].

**Fig. 1:**
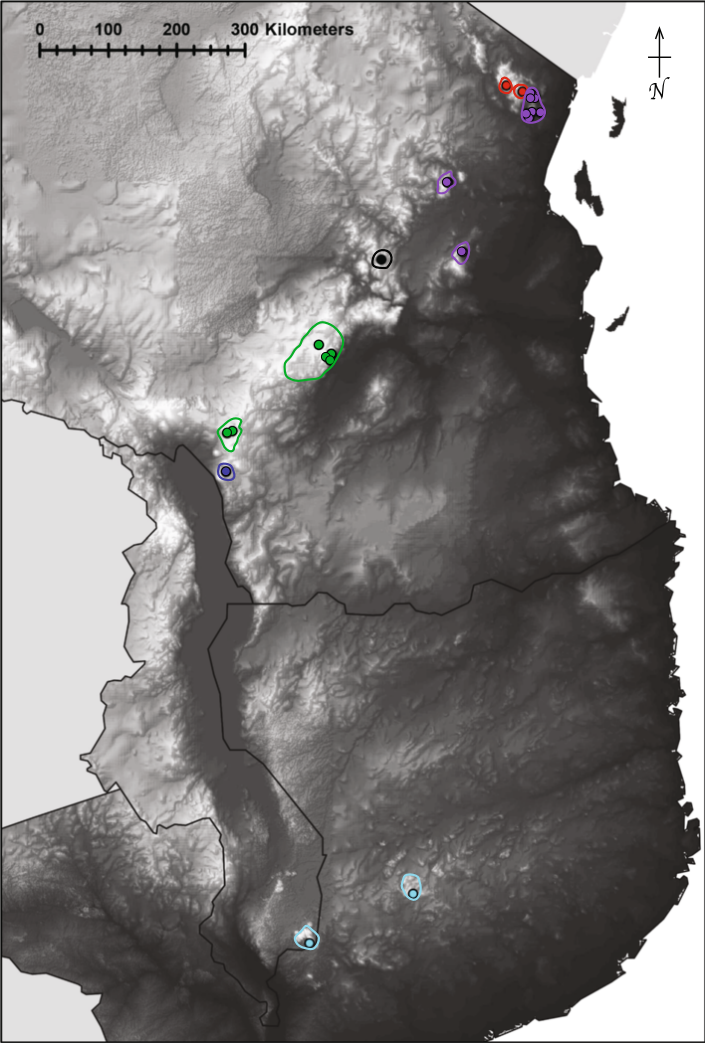
Elevation Map of Kenya, Tanzania, and Malawi encompassing the entire range of species within this complex. Colored dots correspond to localities sampled for each species in this analysis (GPS in supplementary material): red = *Hyperolius tanneri*, purple = *H. burgessi*, black = *H. ukwiva*, green = *H. minutissimus*, dark blue = *H. davenporti*, light blue = *H. spinigularis*. Estimated IUCN range limits for each species are drawn in corresponding colors

In this study we evaluate the African spiny-throated reed frog complex from the EABH of Tanzania, Malawi, and Mozambique (Fig. 1) and quantitatively assess the isolation mechanisms among sister-lineages. The spiny-throated complex is comprised of six taxa (*H. burgessi, H. davenporti, H. spinigularis, H. tanneri, H. minutissimus, and H. ukwiva*) [45], with species occupying localities often restricted to either montane mosaic or rainforest habitats [26, 46–49]*.* By using molecular, morphological and ecological indicators, we make a first assessment of whether allopatric or peripatric speciation processes are supported and whether peripatric speciation is facilitated by the fluctuating montane forest/grassland mosaic biome. These results highlight a possible speciation syndrome within biodiversity hotspots composed of a variety of habitat types where variation in persistence may generate endemicity.

## Results

### Phylogenetic relationships

Summary statistics on the loci used in this study are shown in Table 1. All molecular sequences are deposited along with voucher numbers in GenBank and are available along with all specimen information in Additional file 1: Table S1.

**Table 1:** Summary statistics of molecular loci used in this study

Summary statistics for each locus												
Locus	Aligned BP	Variable sites	Parsimony-informative sites									
ND2	1144	341	219									
Rag1	1275	72	30									
C-myc	1378	71	47									
POMC	625	52	32									
Combined nuclear loci												
Species	Number of samples	Polymorphic sites	Nucleotide diversity	N.D. sd	Theta S	Theta S sd	Theta Pi	Theta Pi sd	Taj D	P Taj D	Fu FS	P FS
*H. burgessi*	21	77	0	0	20.29	7.09	13.73	7.17	-1.37	0.06	-10.76	0
*H. tanneri*	3	25	0.01	0	14.67	9.12	16	12.37	-12799658.42	0	1.63	0.52
*H. minutissimus*	6	39	0	0	17.08	8.35	12.8	7.78	-1.6	0	5.58	0.99
*H. spinigularis*	8	40	0	0	13.11	5.98	10.86	6.3	-1.7	0.02	-0.3	0.35
*H. ukwiva*	1	NA	NA	NA	NA	NA	NA	NA	NA	NA	NA	NA
*H. davenporti*	1	NA	NA	NA	NA	NA	NA	NA	NA	NA	NA	NA
Mitochondrial												
Species	Number of samples	Polymorphic sites	Nucleotide diversity	N.D. sd	Theta S	Theta S sd	Theta Pi	Theta Pi sd	Taj D	P Taj D	Fu FS	P FS
*H. burgessi*	21	27	0.01	0	7.23	2.74	5.42	3.04	-1.06	0.16	0.36	0.6
*H. tanneri*	3	7	0	0	4	2.73	4.67	3.9	0	0.79	0.31	0.37
*H. minutissimus*	6	9	0	0	3.94	2.18	2.33	1.7	-2.43	0	-0.22	0.33
*H. spinigularis*	8	13	0	0	4.63	2.32	3.61	2.33	-1.38	0.09	0.1	0.5
*H. ukwiva*	1	NA	NA	NA	NA	NA	NA	NA	NA	NA	NA	NA
NA	NA	NA	NA	NA	NA	NA	NA	NA	NA	NA	NA	NA

**Fig. 2:**
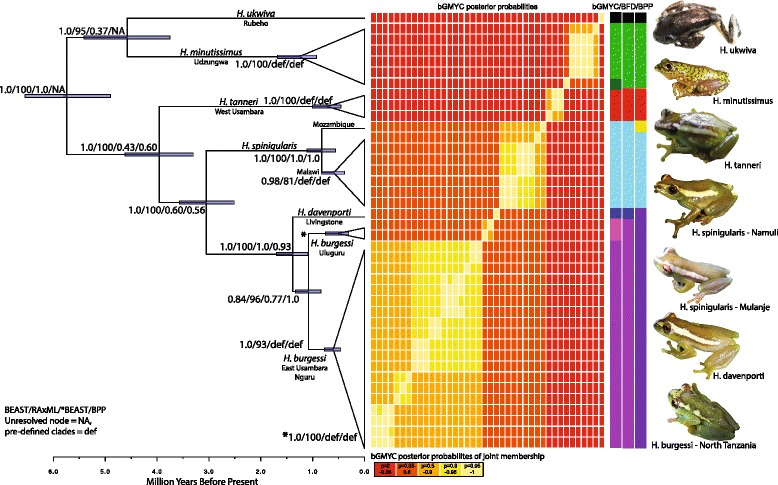
Species trees and species delimitation. Left: Rate calibrated BEAST tree with nodal support values from each species tree and phylogenetic tree method (BEAST/RAxML/*BEAST/BPP). Root age = 5.74 mya. Unresolved node = NA, pre-defined clades (for species tree analyses) = def. Right: bGMYC posterior probabilities of species delimitation are shown as well as simplified species delimitations using bGMYC, BFD, and BPP methods. Each colored block corresponds to a single individual. Pictures from each named species are shown

Species tree methods all agreed on a single reconstruction of the evolutionary relationships within this group, though support for the sister relationship of *H. ukwiva* and *H. minutissimus* was weak or unresolved in some methods (Fig. 2). All methods show monophyletic relationships supporting six lineages: the previously described species of *H. tanneri* and *H. minutissimus*, a new species from the Rubeho Mountains (*H. ukwiva*), three distinct lineages (*H. spinigularis*, *H. davenporti*, and *H. burgessi*) which once were lumped into a single species (*H. spinigularis*) (see [45]). Hereafter the clade previously refered to as *H. spinigularis* will be refered to in quotes (“*spinigularis*” clade) and the newly defined species names will be used. RAxML genetrees are shown in Additional file 2: Figure S1. Specimens previously identified as *H. spinigularis* from the Udzungwa Mountains [48] were included in this analysis and all were definitively genetically and morphologically *H. minutissimus*, confirming earlier estimates that only *H. minutissimus* is found in the Udzungwa Mountain block.

### Species delimitation

Species delimitation methods (*Bayesian General Mixed Yule Coalescent* - bGMYC, *Bayes factor delimitation* - BFD, *Bayesian Phylogenetics and Phylogeography* - BPP) were largely in agreement in defining lineages within this radiation except for some variation in support of additional distinct lineages in addition to the six described here and in [45] (Fig. 2; Table 2 and Additional file 2: Table S2). In each case of additionally distinct lineages associated with the named six (Udzungwa Scarp locality of *H. minutissimus* shown in dark green for bGMYC, Mozambique (Mt. Namuli) specimen for *H. spinigularis* shown in yellow for BPP, Uluguru Mountains locality of *H. burgessi* shown in pink for bGMYC), a distinct locality with comparatively low sampling within a large distribution is involved. Additional sampling will be necessary to determine whether additional taxonomic units are supported. The bGMYC probability threshold above which individuals are considered conspecific was set at 0.5 (equivalent to selecting the posterior mean of the analysis) to avoid over lumping or splitting species (as outlined in the program manual). BFD support for the six-species model shown in Fig. 2 was positive (Path sampling: BayesFactor of 5.4, Stepping Stone BF of 5.8 over the next best model), as was BPP3 (Posterior probability = 0.83410).

**Table 2:** Species Delimitations from BFD *BEAST stepping stone and pathsampling and BPP

*BEAST stepping stone and pathsampling results from BFD					
Species delimitation	Species groups by locality	PS	SS	PS BF	SS BF
All spinigularis	[EU,NG,UL,SH,MU,NA][UD][RU][WU]	-10836.5	-10837.73		
Each population	[EU][NG][UL][SH][MU][NA][UD][RU][WU]	-10813.27	-10813.62	46.46	48.22
All tanzanian spinigularis	[EU,NG,UL,SH][MU,NA][UD][RU][WU]	-10787.96	-10789.17	50.62	48.9
Eastern Arc spinigularis	[EU,NG,UL][SH][MU,NA][UD][RU][WU]	-10782.59	-10783.37	10.74	11.6
BPP species Delimitation from 10,000 iterations					
# of species	Species groups by locality	Groupings	Posterior		
5	[EU,NG,UL,SH][WU][UD][RU][MU,NA]	191	0.02		
6	[EU,NG,UL][SH][WU][UD][RU][MU,NA]	39	0		
6	[EU,NG,UL,SH][WU][UD][RU][MU][NA]	8302	0.83		
7	[EU,NG,UL][SH][WU][UD][RU][MU][NA]	1467	0.15		
8	[EU,NG][UL][SH][WU][UD][RU][MU][NA]	1	0		

Areas are coded by mountain block as follows: EU = East Usambara, NG = Nguru, UL = Uluguru, SH = Southern Highlands, MU = Mu, NA = Namuli, UD = Udzungwa, RU = Rubeho, WU = West Usambara. BFD: Log likelihood values (lnL) are given for potential species grouping scenarios for path sampling (PS) and stepping stone analysis. Bayes Factors of each progressively more likely scenario are shown (BF). Bayes Factors above 10 are considered “decisive”. Top models from each analysis both predict six species, though BPP lumps *H. davenporti* and *H. burgessi* and splits the two populations of *H. spinigularis*

### Effective population size, population bottlenecks and expansions

**Fig. 3:**
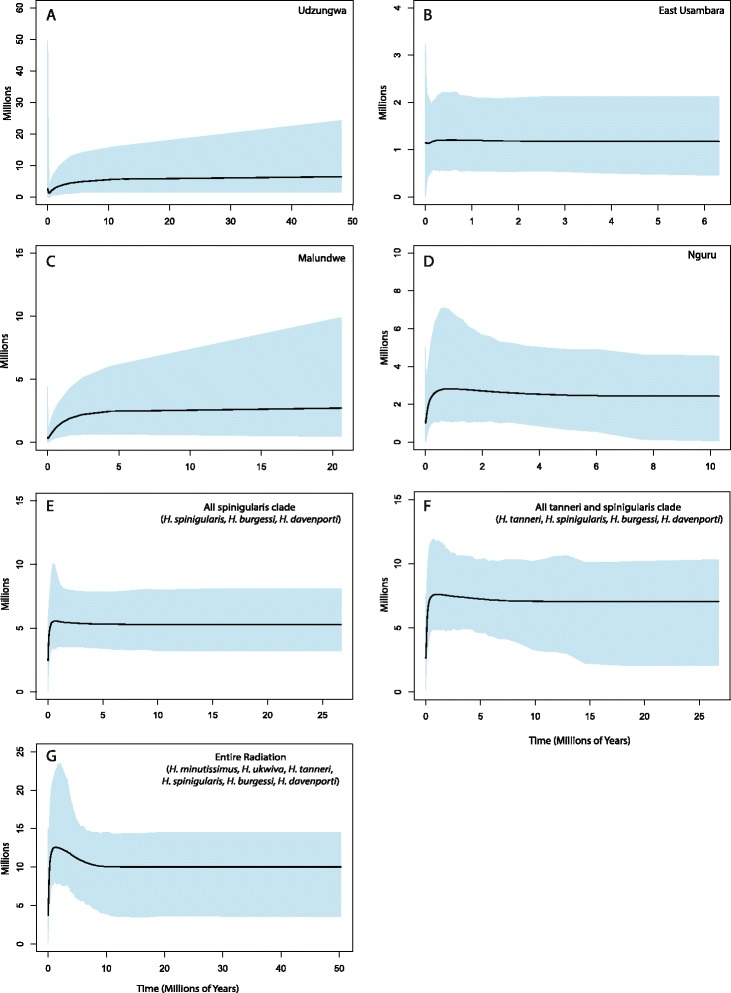
EBSP plots of effective population sizes and potential bottlenecks in populations and species with largest sample sizes. **a**-**d** show single mountain block populations within species. **a** *H. minutissimus* population in the Udzungwa Mountains. **b** *H. burgessi* in the East Usambara Mountains. **c** *H. burgessi* on Malundwe Hill. **d** *H. burgessi* in the Nguru Mountains. **e**-**g** show all descendent individuals from deeper phylogenetic nodes

No populations showed signs of ancestral bottlenecks or expansions, though the narrowly distributed species (*H. tanneri*, *H. ukwiva*, *H. davenporti*; distributions in Table 3) did not have sufficient sample sizes to analyze Extended Bayesian Skyline Plots (EBSP). In EBSP plots, most populations showed potential current bottlenecks, which may be related to recent range reductions of habitats due to climatic and/or anthropogenic events (Fig. 3). Only the population of *H. burgessi* in the East Usambara Mountains does not appear to show a current reduction, though this is also the population with greatest sampling. EBSP plots of all individuals from deeper nodes did not show bottlenecks or expansions except when including all individuals from all species, which shows an expansion beginning around 8 MYA that peaks around 4 MYA. This coincides with the split between *H. tanneri* and the *H. spinigularis/burgessi/davenporti* clade.

**Table 3:** Species, altitudinal range, habitat and available area of occurrence from IUCN estimates

Species	Altitudinal range	Habitat	Expected occurrence
*H. burgessi*	East Usambara: 900–1100 m	Submontane forest	14,774 km^2^
	Nguru: 900–1000 m		
	Uluguru: 980 m		
*H. davenporti*	Livingstone: 2010 m	Montane forest edge	28 km^2^
*H. minutissimus*	Njombe: 2010 m	Montane forest edge and grassland	14,904 km^2^
	Udzungwa: 1680–1970 m		
*H. spinigularis*	Malawi: 690 m	Submontane forest and forest edge	5,488 km^2^
	Mozambique: 1250 m		
*H. tanneri*	West Usambara: 1310–1650 m	Submontane forest and forest edge	4 km^2^
*H. ukwiva*	Rubeho: 1660 m	Montane forest edge	1,179 km^2^

BPP estimates of Theta (θ) show essentially stable mean and 95 % confidence intervals for all splits within the tree (e.g., θ from median gamma prior across nodes: ~ 0.003 (~0.0007, 0.007)). However, estimates from the limited-distribution species are based on 1–3 individuals, and thus are not informative. Stable θ estimates of the species with larger distributions (and corresponding divergence nodes) agree with EBSP population estimates that these populations have been stable through time without marked reductions or expansions (Additional file 2: Figure S2).

### Morphological divergence

**Fig. 4:**
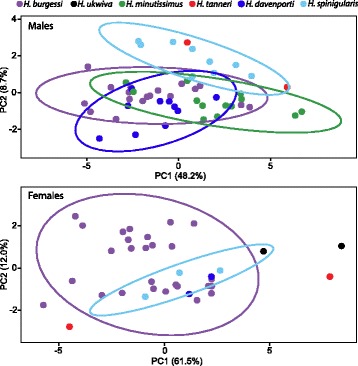
Principle Component Analysis of morphological divergence between species. Males are distinguished by Snout-Urostyle length (SUL) and aspects of Gular shape. Females are distinguished by SUL

Measurements for all traits and species summary statistics are presented in [45]. In males, aspects of gular shape (height, width) contributed most to distinguishing species in PC1 (48 %, *p* = 0.001) (Fig. 4, Additional file 2: Table S3), though overall size (snout-urostyle length, SUL) was also significantly involved. PC2, though contributing much less to distance between the groups, separates *H. tanneri* and *H. spinigularis* from all other species based on head length and gular flap height. Both gular shape estimates and SUL were significant in single trait ANOVA estimates (Table 4). For females, head-width, SUL, and leg measurements were the primary distinguishing characteristics in PC1 (62 %, *p* = 0.01). In ANOVA analyses of these traits controlled for size, only SUL was significantly different between species (Table 4, [45]). Presence and distribution of gular and ventral spines are different between all species [45] and is a diagnostic tool for separating species (see [45]).

**Table 4:** ANOVA and Kruskal-Wallis (KW) analyses of morphology by species

Females	Df	Anova sum Sq	Anova mean Sq	Anova F	Anova Pr(>F)	KW chi-sq	KW P
SUL	4	0.079	0.020	5.973	0.001	9.783	0.044
SUL Residuals	32	0.105	0.003				
TL/SUL	4	0.029	0.007	1.766	0.160	6.822	0.146
TL/SUL Residuals	32	0.131	0.004				
HW/SUL	4	0.002	0.001	0.286	0.885	2.119	0.714
HW/SUL Residuals	32	0.065	0.002				
Males	Df	Anova sum Sq	Anova mean Sq	Anova F	Anova Pr(>F)	KW chi-sq	KW P
SUL	4	0.116	0.029	7.941	0	17.968	0.001
SUL Residuals	48	0.176	0.004				
TL/SUL	4	0.013	0.003	1.641	0.179	7.307	0.120
TL/SUL Residuals	48	0.094	0.002				
HW/SUL	4	0.037	0.009	1.962	0.115	8.294	0.081
HW/SUL Residuals	48	0.224	0.005				
WGF/SUL	4	0.126	0.032	5.736	0.001	17.323	0.002
WGF/SUL Residuals	47	0.258	0.005				
HGF/SUL	4	0.816	0.204	51.880	<2e-16	40.704	3.10E-08
HGF/SUL Residuals	47	0.185	0.004				
WGF/HGF	4	0.905	0.226	84.290	<2e-16	43.907	6.71E-09
WGF/HGF Residuals	47	0.126	0.003				

Morphological measurement abbreviations are: Snout-Urostyle length (SUL), Tibiafibula Length (TL), Head Width (HW), Gular Flap Width (GFW), Gular Flap Height (GFH). For raw measurements, see Loader et al., submitted

### Environmental niche divergence, timing of shifts, and range sizes

**Fig. 5:**
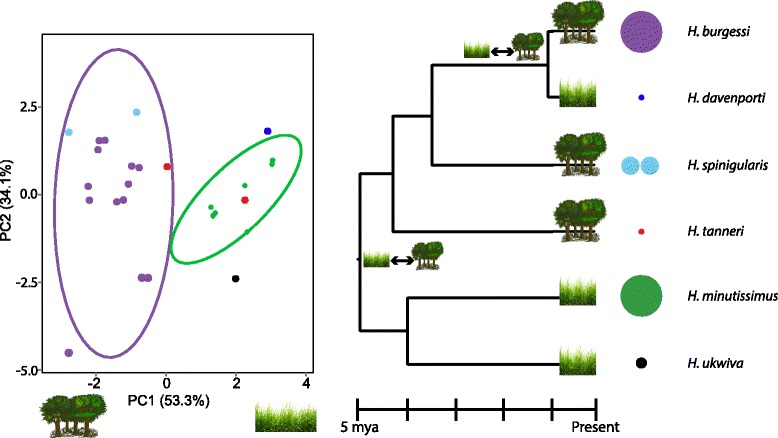
Ecological Niche Divergence of speciation according to major habitat types. Left: Principle Component Analysis of current habitat suitability for each species. Montane grassland/forest mosaic species are on the right, while rainforest adapted species are on the left. Right: Distribution of forest (trees) and mosaic (grasses) adapted species on *BEAST species tree. A double-sided arrow between habitat types indicates the two nodes where ecological shifts are inferred. Relative population sizes based on field collection estimates and potential range sizes are shown in the relative size of color-key circles. The split distribution of *H. spinigularis* is represented by two circles to represent the two distant populations in Malawi and Mozambique separated by ~160 km. The two localities of *H. tanneri* are represented by a single circle as they are within the same mountain block and separated by only ~25 km

PCA analysis of full and reduced bioclim variables (19 vs. 7) showed equivalent patterns, and thus only the reduced dataset is discussed. Two major clades of species were identified along PC1 marking the split between a rainforest adapted group (*H. burgessi*, *H. tanneri*, and *H. spinigularis*) and a mosaic-adapted group (*H. ukwiva*, *H. minutissimus*, and *H. davenporti*) (non-overlapping 95 % confidence intervals; Fig. 5, Additional file 2: Table S4). PC1 (53 % explained variance) is dominated by the temperature and precipitation during the coldest and driest times of year. These habitat syndromes, when mapped onto the phylogenetic tree, show two major adaptive habitat shifts to high elevation grasslands (Fig. 5), presuming that the ancestor was forest-adapted in line with paleoclimatic estimates of conditions ~ 4 MYA [50], before a general shift to grasslands at 1.86 MYA [51].

In testing potentially peripatric sister species pairs for niche shifts, *H. davenporti* falls outside of the PCA confidence ellipse probability threshold for *H. burgessi* into different niche space (Fig. 5). The presence of *H. davenporti* and *H. burgessi* in two divergent areas of niche space (mosaic vs. rainforest) implies a major niche shift at the time of speciation. *Hyperolius ukwiva* localities were outside of the PCA confidence ellipse probability threshold for *H. minutissimus* (Fig. 5), but only separated by PC2. The appropriate comparison for *H. tanneri* in a phylogenetic context is between *H. tanneri* and the ancestor of *H. burgessi*, *H. spinigularis*, and *H. davenporti*. To approximate their ancestral niche, we combined distributional data from these three species into a lumped distribution, which we then compared to *H. tanneri* in a PCA analysis as above. No separation was seen between *H. tanneri* and the combined niche space of the “*spinigularis*” group (*H. burgessi*, *H. spinigularis*, and *H. davenporti*) (Additional file 2: Figure S3).

The two cases of adaptation to grasslands (basal split and *H. burgessi*/*H. davenporti*) occurred at differing times (Fig. 5, ~5 MYA and ~ 1 MYA) and therefore a single event cannot have caused these shifts. Both events coincide with periods of increased climatic cycling and a gradual increase in coverage from the forest/grassland mosaic from the previously extensively distributed rainforests of this region. Geographic range estimates (Table 3), underline range asymmetries between sister species [52].

## Discussion

### New lineages within the spiny-throated reed frog clade

Prior to this study and our complementary taxonomic revision [45], only three species were recognized within the spiny-throated reed frog radiation within the highlands of East Africa: *H. spinigularis, H. tanneri*, and *H. minutissimus*. Our field surveys in isolated mountain ranges and associated molecular analyses and morphological assessments of members within this group have documented three new lineages within this radiation: *H. burgessi, H. davenporti*, and *H. ukwiva*, each corresponding to a different mountain block or blocks within the EABH. In addition to morphological diagnoses, species delimitation methods based on molecular data were in general agreement in the recognition of each of these species. Deeper population-level sampling should help clarify the levels of divergence within and between species. Nodal support was strong in most methods, though *BEAST showed weak support at basal nodes. Increased sampling of additional molecular markers may help clarify relationships in these deeply divergent lineages.

### Peripatric speciation or not

Three species within this radiation are known from single localities in the mountains of the EABH (*H. tanneri*, *H. davenporti*, *H. ukwiva*), raising the question of whether these lineages are the result of peripatric speciation events when isolated from their broadly distributed sister species. Though evidence is limited, many cases of speciation within the spiny-throated reed frogs are consistent with peripatric speciation. In support of peripatric origins, the three species with limited distributions all have smaller realized and potential distributions than their broadly distributed sister lineages (Table 3). Uneven population sizes and population bottlenecks could not be explicitly tested in peripatric species as field observations showed very low population densities and genetic material was limited, yet the rarity of populations and low population sizes of the limited-distribution species were apparent from repeated surveys across localities [53]. In contrast, their sister lineages showed much larger distributions with large and stable population sizes through time. Minimally, the lack of bottleneck patterns from the well sampled, broadly distributed species and limited distribution of sister lineages fits with our *a priori* predictions for peripatric speciation.

We expected peripatric lineages would differ in climate/habitat, and this was found in 2/3 pairs. Though a habitat shift is not necessary for peripatric speciation, these shifts are less likely in vicariant speciation. The expectation of accentuated divergence in sexually-dimorphic traits in peripatric lineages compared to allopatric was not altogether supported, as all species differed in SUL, gular shape, and the arrangement of gular spines which we assume to be involved in some aspect of sexual selection. There is, however, a complete loss of gular spines in *H. tanneri* differing substantially from its sister group. This rapid morphological change in *H. tanneri*, potentially the result of sexual selection, is what would be predicted for peripatric speciation.

Within the allopatric populations of both *H. burgessi* and *H. spinigularis*, we find conserved ecological niche/environmental tolerance despite population distances greater than most peripatric species pairs, and no evidence of population bottlenecks. As most species within this radiation are known from single mountain blocks, we were concerned that morphological variation would be high even within species on different mountain blocks (*H. spinigularis* and *H. burgessi* have distributions spanning multiple mountain blocks). However, this was not the case in *H. burgessi*, where morphology was conserved despite substantial divergence times (ca. 0.3-1 mya) between different blocks. Sufficient morphological data are not yet available for the newly discovered Mozambique population of *H. spinigularis* to determine if they are similarly conserved in relation to Malawian specimens.

Distinguishing between allopatric and peripatric speciation can be difficult, as these processes anchor a continuum of progressively asymmetric isolation. The precise point at which a process ceases to be allopatric and becomes peripatric is therefore blurred. The cases of mixed support in the indicators we examined might therefore be a consequence of the extent to which some of the events were truly “peripatric” or “allopatric” events. Likewise, the indicators themselves (current range sizes, potential ecological niche shifts, and accelerated morphological diversification) may yield ambiguous results when evaluating species pairs that are deeply divergent. With the current data available on the spiny-throated reed frogs, it seems possible that peripatric speciation plays a role in generating biodiversity within the forests and grasslands of the EABH. In systems such as the EABH, which contain a high concentration of old endemics with limited and fragmented distributions in isolated highlands [34], a community approach may be necessary to clarify the prevalence of these speciation mechanisms in order to more fully understand the formation of its rich biota.

### Biome specific speciation mechanisms

Based on the comparisons outlined here from spiny-throated hyperoliids found across these habitats – it seems that peripatric processes may be more pronounced in higher elevation species compared to those in more persistent rainforests (e.g., [41]). If there were a correlation between the less persistent montane forest/grassland mosaic sites and peripatric speciation, we would expect a common biogeographic pattern repeated across other species. Little data from other amphibians of peripatry has been reported worldwide (e.g., [41, 54, 55]), however, and information is particularly lacking for amphibians within the EABH. Similarly little is known about the phylogenetic relationships and demographic history among EABH high-altitude species and whether patterns exist that might be consistent with peripatry. Evidence in analogous systems, however, suggests that such a distinct boundary in biomes – as in rainforest and grasslands - might be important for generating specific speciation mechanisms. For example, ecotones across Savanna-Forest mosaics of Central Africa been suggested to be important in generating rainforest diversity through peri – and para – patric processes [56]; however, the generality of such patterns has not be quantified. The role of distinct habitat boundaries promoting speciation processes clearly deserves further investigation, potentially such situations might favor fundamentally different speciation processes compared to other areas (such as vicariance within lower elevation forest habitats). Likewise, there are also clear conservation implications that arise from these considerations. If high elevation species consistently exhibit limited population numbers and historical population genetic bottlenecks, they may be at increased risk if exposed to global anthropogenic pressures such as habitat destruction, fragmentation, and climate change.

## Conclusions

Molecular, morphological, range, and niche analyses provide mixed support for cases of peripatric speciation in spiny-throated reed frogs. The highest support is in species pairs exhibiting extreme potential and realized distributional asymmetry, niche shifts, and morphological divergence driven by sexually selected traits: *H. burgessi* vs. *H. davenporti*; *H. minutissimus* vs. *H. ukwiva*. The older break between *H. tanneri* and the “*spinigularis*” clade of *H. spinigularis/burgessi/davenporti* is less clear, though the limited distribution and unique loss of gular spines (in this clade) in *H. tanneri* both support the potential for a peripatric split across the 10 km gap separating their current distributions. Though low survey numbers and extremely limited range size in potentially peripatric populations limited our ability to employ demographic reconstructions to confirm peripatric speciation from a signature of founding bottlenecks within the narrowly-distributed species, these conditions themselves support that these are small and divergent populations on the periphery of sister lineages with larger geographic distributions. The emergence of the highland forest/grassland mosaic ecosystems during the same historical interval of this radiation may have facilitated a peripatric mode of speciation as these novel habitats created dispersal opportunities within the larger ranges of rainforest adapted species. Whether the high elevation species that occur along the edge of forest and grassland areas are more amenable to peripatric speciation than rainforest-restricted species awaits additional assessment across more taxonomic groups. However, biome specific peripatric speciation process appears to be supported in some EABH hyperoliids.

## Methods

### Molecular data

We sampled 40 individuals (*Hyperolius spinigularis* = 8, *H. tanneri* = 3, *H. burgessi* = 21, *H. ukwiva* = 1, *H. minutissimus* = 6, *H. davenporti* = 1) from nine mountain blocks, covering the entire range of the spiny-throated reed frog complex (Fig. 1, Sample sizes, Museum numbers and GPS coordinates in Additional file 1: Table S1). Outgroup members of the genus *Hyperolius* and the family *Hyperoliidae* were also collected from the field. We collected full voucher specimens, tissue samples, photographs, and microhabitat information. All specimens for this study were collected in accordance with animal ethics guidelines established in the institutions of authors (including Field Museum of Natural History, Science Museo of Trento, and University of Basel).

We extracted total DNA from liver and leg muscle tissue of freshly collected specimens preserved in 95 % ethanol or 20 % DMSO buffer [57] using the PUREGENE DNA Purification Kit protocol (Qiagen, Valencia, CA). We sequenced one mitochondrial and three nuclear loci: (1) mitochondrial ND2 gene and flanking tRNAs (NADH dehydrogenase subunit 2: 1144 bp), (2) POMC (Pro-opiomelanocortin: exon 629 bp), (3) C-myc (cellular myelocytomatosis proto-oncogene: exons and intron 1335 bp), and (4) Rag-1 (recombination activating gene: exon 1282 bp). Primers are the same as in Lawson (2010) with the addition of Rag-1 primers designed for this study (Table 5, [58]). Extraction, amplification, sequencing, and cloning of alleles follow Lawson (2010) [26].

**Table 5:** Primers use in this study

Primer	Sequence	Origin
Cmyc 1U	GAGGACATCTGGAARAARTT	Crawford 2003 [61]
Cmyc 3L	GTCTTCCTCTTGTCRTTCTCYTC	Crawford 2003 [61]
Cmyc H int	GAACAGCTTGACATGCAGTAC	Lawson 2010 [26]
Cmyc L int	CTGCTCAGATTGGTCTACAGC	Lawson 2010 [26]
POMC1	GAATGTATYAAAGMMTGCAAGATGGWCCT	Wiens et al. 2005 [59]
POMC2	TAYTGRCCCTTYTTGTGGGCRTT	Wiens et al. 2005 [59]
ND2–H Trp	GCTTTGAAGGCYKTTGGT	Lawson 2010 [26]
ND2–L Gln	GTTCAAACCCCMTCACTTCCT	Lawson 2010 [26]
rag1.for	GCCAGATCTTTCARCCACTC	This study – designed internal to Hoegg et al. 2004 [58]
rag1.rev	TGATCTCTGGAACRTGGGCTA	This study – designed internal to Hoegg et al. 2004 [58]

Alignments were performed using MUSCLE [60] as described in [26]. Alignments were unambiguous in ND2, POMC, and Rag-1, and easily aligned in C-myc despite the indel-prone intron region. All protein-coding regions were translated into amino acids to check for errors. Substitution rates for each locus are described in Lawson (2010) [26]: ND2 0.00957/lineage/my [61]; C-myc 0.0006334/lineage/my [26]; POMC 0.000721/lineage/my [26]; and Rag-1 0.00042/lineage/my [62], and used for approximate estimates of diversification events. One nuclear allele from each individual (either cloned or phased as outlined above) was randomly discarded to create a 1:1 mtDNA:nuDNA dataset. Summary information for each locus shown in Table 1.

### Phylogenetic reconstruction and divergence time estimation

Sequences from [26] and 10 additional East African *Hyperolius* and *Afrixalus* were included in initial analyses to assess monophyly of the spiny-throated reed frogs (Additional files) including an additional proposed close relative, *H. parkeri* [63], using phylogenetic methods described below. A number of datasets were used for initial assessment of phylogenetic relationships: species tree (single representative per species and all outgroups), population/species tree (all spiny-throated specimens (ingroup) and all outgroups), and population-level analysis of only spiny-throated specimens. Monophyly of the ingroup individuals was strongly supported in all cases. Final Bayesian inference (BI) and maximum likelihood (ML) phylogenetic analyses were then completed with the full ingroup dataset (all spiny-throated specimens). RAxML (ML) and BPP (BI) analysis included a single arbitrary close outgroup (*H. mitchelli*), while the BEAST (BI) and *BEAST (BI) rate-calibrated trees were completed without an outgroup for improved precision of branch length estimates. RAxML (version 7.0.4) [64] analyses used the rapid hill climbing algorithm and the GTRGAMMA substitution model [65] partitioned by gene and codon. BEAST (versions 1.8 and 2.1.3) [66] and *BEAST (starBEAST) [67], were partitioned by locus and codon position (SRD06 model) [68]. BPP3 [69, 70] parameters listed below in species delimitation. For both ML and BI analyses, model parameters were independently optimized for each partition. All analyses were run for 10 million generations, and run twice to ensure stability (results not combined). The first 10 % of total generations were discarded as burnin for both convergence and tree estimates. Convergence was investigated using Tracer (version 1.6) [71] through a visual inspection of adequate mixing and ESS estimates >200. The maximum clade credibility tree was calculated for BEAST and *BEAST trees using TreeAnnotator in BEAST. ML node support was evaluated by non-parametric bootstrapping [72] with 1000 replicates performed in RAxML. The Majority Rule Consensus tree from BPP3 is reported from Evolver in PAML 4 [73].

BEAST analysis was run with a coalescent, constant size tree-prior and a strict molecular clock (as recommended for recent population-level analyses). *BEAST and BPP3 analysis used individuals from the same mountain block as discrete units (after confirmation of monophyly from individual-based tree constructions) with the exception of a single unit containing individuals from the East Usambara Mountains and Nguru Mountains which were not mutually monophyletic. A Birth-Death tree prior was used in *BEAST due to *a priori* hypotheses that this lineage has likely undergone local population extinctions through its history.

### Species delimitation

The program BPP3 [69, 70] was used to jointly infer the species tree and species limits. Default priors and settings were used: gamma prior = G(2, 1000) with mean 2/2000 = 0.001 for population size parameters (s, θ); gamma prior G(2, 1000) for the species tree root age (τ0); all other divergence time parameters are assigned the Dirichlet prior [70] equation two). Analyses were also run with a very large (1,10) and a very small mean (1, 10000) for θ and τ0 to ensure stability of results. A heredity file (G(4,5)) and locus rate file were incorporated to account for the combined mitochondrial and nuclear datasets and associated mutation rates. Each analysis was run twice to confirm consistency between runs. Species delimitation was assessed with rjMCMC algorithm 0, e = 2.5.

To establish species boundaries within the spiny-throated reed frogs, a Bayesian General Mixed Yule-Coalescent (bGMYC) model was implemented in the package bGMYC [74] in R v. 3.0.3 [75] using 100 random trees from the concatenated BEAST. Simulations were set at 50,000 generations with 40,000 burn-in, sampling every 100th generation. The upper threshold for the number of species was set at 40, to match the number of tips on the tree.

Bayes Factor species Delimitation (BFD; [76]) was used to compare three alternative species scenarios using stepping stone and pathsampling analysis in *BEAST summarized in Table 2. Based on the depth of nodes and morphological divergence, *H. tanneri*, *H. minutissimus*, and *H. ukwiva* are all considered separate species in all analyses. All *BEAST parameters are as above. These approaches were used to define units for analyzing molecular, morphological and ecological parameters among lineages.

### Effective population size

BPP3 was used to estimate coalescent-scaled population sizes (θ = 4Neμ) and time of divergence (τ = μ t) throughout this radiation on the fixed species tree recovered from all phylogenetic analyses. The parameters used are the same as in the joint species delimitation and species tree analysis.

To test for signs of demographic expansion or contraction we implemented multilocus extended Bayesian skyline plots (EBSP) in BEAST v. 1.8 on populations with n > 5 (Mulanje Massif, Malawi; East Usambara Mountains, Tanzania; Nguru Mountains, Tanzania; Udzungwa Mountains, Tanzania) and all individuals within the “*spinigularis*” clade to calculate population size through time and the probability of bottlenecks or expansion. All EBSP analyses used a strict clock. Operators were modified according to author recommendations and analyses were run for 10 million generations to obtain adequate ESS values.

### Morphological divergence

To determine morphological distinctiveness among members of the species cluster, we took commonly-used limb and cranial measurements for 17 traits [77], including leg bone (e.g. femur, tibia) and foot lengths and head width (see below). Many of these traits are often assumed to be adaptive, as they are correlated with food source (*e.g.,* head width), perching (*e.g.,* foot length), and other life history traits. If species are morphologically divergent, it is possible that competition or adaptation to different environmental conditions reinforced separation within this radiation. Morphological measurements made in this analysis include: Snout-Urostyle Length (SUL), Head Width (HW), Head Length Diagonal from corner of mouth (HLD), Head Length Diagonal from jawbone end (HLDJ), Nostril-Snout (NS), Inter-narial (IN), Eye to Nostril (EN), Eye Distance (EE), Inter-orbital (IO), Tibiafibula Length (TL), Thigh Length (THL), Tibiale Fibulare Length (TFL), Foot Length (FL), Forelimb Length (FLL), Hand Length (HL), Width of Gular Flap (WGF) Height of Gular Flap (HGF). We also scored specimens for traits associated with sexual selection including presence and distribution of gular spines (which are only present during the breeding season for species that possess them) and color patterns. Morphological measurements were taken of 114 mature specimens using Mitutoyo Absolute Digimatic Calipers (CD-6"C) (Sample sizes and Measurements in supplementary materials). In order to assess the morphological distinctness of these species, we conducted Principal Component analyses on log-transformed data of males and females separately with all variables centered and scaled in R [78] (95 % confidence ellipse probability threshold from ggbiplot package). To further evaluate whether species were significantly different, we did a Permutational Multivariate Analysis of Variance Using Distance Matrices using the adonis function from the vegan [79] R package.

Kruskal–Wallis and ANOVA were used to assess variable divergence (standardized by body size) between species for use as diagnostic characters in R.

### Niche divergence

Niche similarity of all species was assessed by Principle Component Analysis (PCA) using bioclim variables associated with GPS coordinates (Additinal file 1: Table S1) in the MASS and ggbiplot packages in R (95 % confidence ellipse probability threshold). This method allows at least a preliminary view of habitat similarity for severely range-restricted taxa such as the members of this complex. We evaluated both a full bioclim dataset (all 19 standard bioclim variables) and a reduced dataset with Pearson’s correlation coefficients below 0.7: Mean Diurnal Range, Temperature Seasonality, Temperature Annual Range, Mean Temperature of Coldest Quarter, Precipitation of Wettest Month, Precipitation Seasonality, Precipitation of Driest Quarter, Precipitation of Warmest Quarter, Precipitation of Coldest Quarter (30 arc-second resolution, WorldClim database [80]). In reconstructing ecological shifts across the phylogenetic tree, we rely on the assumption that a species niche remains stable through time after divergence. Though this appears to be widely true [16, 81, 82], there is increased uncertainty associated with assessing extremely locally adapted lineages were spatial autocorrelation might have a more pronounced effect.

### Range estimates and field observations of population densities

Species’ range estimates were calculated using standardized approaches conducted on species being evaluated for the IUCN Red List of Threatened Species™ [52]. Estimates of population size were made using molecular techniques when sufficient numbers of individuals were available (above). For those species with very few individuals ever found (*H. tanneri*, *H. ukwiva*, *H. davenporti*), estimates were qualitatively assessed from sampling effort over multiple field seasons (Lawson, Menegon, Loader, personal observations, [53]) in comparison to all known localities from other spiny-throated reed frogs (*H. spinigularis*, *H. burgessi*, *H. minutissimus*).

## Additional files

Additional file 1: Table S1. List of specimens analyzed including locality and genbank accession numbers for all loci. Additonal file 2: Figure S1. Maximum Clade Credibility Gene Trees from BEAST. Posterior probabilities shown on relevant nodes. **Figure S2.** Species tree with BPP3 posterior theta values shown for species splits and current populations: Mean (95 % interval) with three theta priors. Top (large mean) = (1, 10). Middle = (2, 1000). Bottom (very small mean) = (1, 10000). Populations IDs match Table 2. **Figure S3.** PCA analysis of bioclim variables with all members of the “*spinigularis*” clade (*H. burgessi*, *H. davenporti*, *H. spinigularis*) combined to contrast from *H. tanneri*. *Hyperolius tanneri* specimens do not both fall outside of the 95 % confidence interval for the *H. spinigularis* members, thus no habitat shift is inferred. **Table S2.** Summary of BPP species delimitation results from 10000 delimitations. Populations were defined as: all individuals from East Usambara and Nguru Mountains (Eung), Uluguru Mountains (Ul), Southern Highlands (SH), West Usambara Mountains (Wu), Udzungwa Mountains (Ud), Rubeho Mountains (Ru), Mulanje Massif (Mu), Mount Namuli (Na), and the outgroup *Hyperolius mitchelli* (Mit). **Table S3.** PCA analyses of morphology for Females and Males. **Table S4.** PCA from reduced set of Bioclim variables. **Table S5.** jModeltest scores for each locus. Top 8 models from each analysis shown. 24 models considered to facilitate utilization in BEAST. Selected models are in bold and outlined.
