# Supplementary material for: Divergence at the edges: peripatric isolation in the montane spiny throated reed frog complex
Source: BMC Evol Biol. 2015 Jul 1;15:128. doi: 10.1186/s12862-015-0384-3 (PMC4487588; doi:10.1186/s12862-015-0384-3)
Supplement: Additonal file 2: Figure S1. — Maximum Clade Credibility Gene Trees from BEAST. Posterior probabilities shown on relevant nodes. Figure S2. Species tree with BPP3 posterior theta values shown for species splits and current populations: Mean (95 % interval) with three theta priors. Top (large mean) = (1, 10). Middle = (2, 1000). Bottom (very small mean) = (1, 10000). Populations IDs match Table 2. Figure S3. PCA analysis of bioclim variables with all members of the “spinigularis” clade (H. burgessi, H. davenporti, H. spinigularis) combined to contrast from H. tanneri. Hyperolius tanneri specimens do not both fall outside of the 95 % confidence interval for the H. spinigularis members, thus no habitat shift is inferred. Table S2. Summary of BPP species delimitation results from 10000 delimitations. Populations were defined as: all individuals from East Usambara and Nguru Mountains (Eung), Uluguru Mountains (Ul), Southern Highlands (SH), West Usambara Mountains (Wu), Udzungwa Mountains (Ud), Rubeho Mountains (Ru), Mulanje Massif (Mu), Mount Namuli (Na), and the outgroup Hyperolius mitchelli (Mit). Table S3. PCA analyses of morphology for Females and Males. Table S4. PCA from reduced set of Bioclim variables. Table S5. jModeltest scores for each locus. Top 8 models from each analysis shown. 24 models considered to facilitate utilization in BEAST. Selected models are in bold and outlined. [file 12862_2015_384_MOESM2_ESM.docx]

Supplementary Fig. S1: Maximum Clade Credibility Gene Trees from BEAST. Posterior probabilities shown on relevant nodes.


Supplementary Figure S2: Species tree with BPP3 posterior theta values shown for species splits and current populations: Mean (95% interval) with three theta priors. Top (large mean) = (1, 10). Middle = (2, 1000). Bottom (very small mean)= (1, 10000). Populations IDs match Table 2.

Supplementary Figure S3: PCA analysis of bioclim variables with all members of the “*spinigularis*” clade (*H. burgessi*, *H. davenporti*, *H. spinigularis*) combined to contrast from *H. tanneri*. *Hyperolius tanneri* specimens do not both fall outside of the 95% confidence interval for the *H. spinigularis* members, thus no habitat shift is inferred.

Supplementary Table S2: Summary of BPP species delimitation results from 10000 delimitations. Populations were defined as: all individuals from East Usambara and Nguru Mountains (Eung), Uluguru Mountains (Ul), Southern Highlands (SH), West Usambara Mountains (Wu), Udzungwa Mountains (Ud), Rubeho Mountains (Ru), Mulanje Massif (Mu), Mount Namuli (Na), and the outgroup *Hyperolius mitchelli* (Mit).

(A) 5 species delimitations & their posterior probabilities

191 0.01910 6 (EungUlSH Wu Ud Ru MuNa Mit)

39 0.00390 7 (EungUl Wu Ud Ru MuNa SH Mit)

8302 0.83020 7 (EungUlSH Wu Ud Ru Mu Na Mit)

1467 0.14670 8 (EungUl Wu Ud Ru Mu SH Na Mit)

1 0.00010 9 (Eung Wu Ul Ud Ru Mu SH Na Mit)

(B) 12 delimited species & their posterior probabilities

1 0.00010 Eung

1506 0.15060 EungUl

8493 0.84930 EungUlSH

10000 1.00000 Wu

1 0.00010 Ul

10000 1.00000 Ud

10000 1.00000 Ru

9770 0.97700 Mu

230 0.02300 MuNa

1507 0.15070 SH

9770 0.97700 Na

10000 1.00000 Mit

(C) Posterior probability for # of species

P[6] = 0.01910

P[7] = 0.83410

P[8] = 0.14670

Supplementary Table S3: PCA analyses of morphology for Females and Males

|  |  |  |  |  |  |  |  |  |
| --- | --- | --- | --- | --- | --- | --- | --- | --- |
| **PCA Females** | PC1 | PC2 | PC3 | PC4 | PC5 |  |  |  |
| Standard dev.: | 3.038 | 1.343 | 0.993 | 0.834 | 0.759 |  |  |  |
| Rotation: |  |  |  |  |  |  |  |  |
| SUL | 0.292 | 0.048 | -0.123 | 0.304 | -0.103 |  |  |  |
| HW | 0.310 | -0.099 | 0.029 | 0.068 | 0.031 |  |  |  |
| HLD_mouth | 0.262 | -0.047 | 0.485 | 0.044 | 0.224 |  |  |  |
| HLD_jaw | 0.278 | -0.112 | 0.310 | 0.240 | 0.085 |  |  |  |
| NS | 0.224 | -0.368 | 0.188 | -0.412 | -0.039 |  |  |  |
| IN | 0.228 | 0.184 | 0.150 | -0.676 | 0.224 |  |  |  |
| EN | 0.224 | -0.453 | -0.010 | 0.225 | -0.082 |  |  |  |
| EE | 0.239 | -0.082 | 0.109 | -0.097 | -0.797 |  |  |  |
| IO | 0.132 | 0.471 | 0.535 | 0.283 | 0.010 |  |  |  |
| TL | 0.291 | -0.135 | -0.178 | -0.100 | 0.126 |  |  |  |
| THL | 0.282 | -0.030 | -0.217 | 0.020 | 0.342 |  |  |  |
| TFL | 0.290 | 0.002 | -0.290 | 0.158 | 0.166 |  |  |  |
| FL | 0.239 | 0.400 | -0.242 | 0.062 | 0.026 |  |  |  |
| FLL | 0.301 | 0.050 | -0.237 | -0.004 | -0.084 |  |  |  |
| HL | 0.222 | 0.434 | -0.140 | -0.192 | -0.263 |  |  |  |
| Importance of components | |  |  |  |  |  |  |  |
|  | PC1 | PC2 | PC3 | PC4 | PC5 |  |  |  |
| Standard dev. | 3.038 | 1.343 | 0.993 | 0.834 | 0.759 |  |  |  |
| Prop. of variance | 0.615 | 0.120 | 0.066 | 0.046 | 0.038 |  |  |  |
| Cummulative prop. | 0.615 | 0.736 | 0.801 | 0.848 | 0.886 |  |  |  |
| **Permutational Multivariate Analysis of Variance Using Distance Matrices** | | | | | |  |  |  |
|  | Df | SumsOfSqs | MeanSqs | F.Model | R2 | Pr(>F) |  |  |
| species | 4 | 1.393 | 0.348 | 3.264 | 0.290 | 0.01 |  |  |
| Residuals | 32 | 3.414 | 0.107 |  | 0.710 |  |  |  |

| **PCA Males** | PC1 | PC2 | PC3 | PC4 | PC5 |  |  |  |  |
| --- | --- | --- | --- | --- | --- | --- | --- | --- | --- |
| Standard dev.: | 2.863 | 1.219 | 1.208 | 1.082 | 1.003 |  |  |  |  |
| Rotation: |  |  |  |  |  |  |  |  |  |
| SUL | 0.273 | 0.147 | -0.276 | 0.099 | -0.251 |  |  |  |  |
| HW | 0.290 | 0.015 | 0.261 | -0.106 | 0.234 |  |  |  |  |
| HLD_mouth | 0.289 | 0.184 | 0.083 | -0.118 | -0.016 |  |  |  |  |
| HLD_jaw | 0.182 | 0.405 | 0.375 | 0.038 | -0.098 |  |  |  |  |
| NS | 0.240 | 0.302 | 0.167 | -0.213 | -0.237 |  |  |  |  |
| IN | 0.213 | -0.285 | 0.287 | -0.179 | 0.176 |  |  |  |  |
| EN | 0.190 | 0.085 | -0.188 | -0.517 | -0.232 |  |  |  |  |
| EE | 0.266 | -0.143 | 0.278 | -0.233 | 0.256 |  |  |  |  |
| IO | 0.170 | 0.016 | 0.305 | 0.600 | -0.121 |  |  |  |  |
| TL | 0.282 | 0.262 | -0.205 | 0.147 | -0.168 |  |  |  |  |
| THL | 0.249 | -0.314 | -0.032 | 0.209 | 0.003 |  |  |  |  |
| TFL | 0.278 | -0.073 | 0.103 | 0.247 | 0.199 |  |  |  |  |
| FL | 0.267 | 0.073 | -0.310 | 0.162 | 0.133 |  |  |  |  |
| FLL | 0.291 | -0.070 | -0.152 | -0.064 | -0.123 |  |  |  |  |
| HL | 0.242 | -0.166 | -0.402 | 0.127 | 0.137 |  |  |  |  |
| GFW | 0.226 | -0.373 | -0.125 | -0.177 | 0.076 |  |  |  |  |
| GFH | 0.028 | -0.480 | 0.205 | 0.018 | -0.726 |  |  |  |  |
| Importance of components | |  |  |  |  |  |  |  |  |
|  | PC1 | PC2 | PC3 | PC4 | PC5 |  |  |  |  |
| Standard dev. | 2.863 | 1.219 | 1.208 | 1.082 | 1.003 |  |  |  |  |
| Prop. of variance | 0.482 | 0.087 | 0.086 | 0.069 | 0.059 |  |  |  |  |
| Cummulative prop. | 0.482 | 0.570 | 0.656 | 0.724 | 0.784 |  |  |  |  |
| **Permutational Multivariate Analysis of Variance Using Distance Matrices** | | | | | |  |  |  |  |
|  | Df | SumsOfSqs | MeanSqs | F.Model | R2 | Pr(>F) |  |  |  |
| species | 4.000 | 2.066 | 0.516 | 5.695 | 0.322 | 0.001 |  |  |  |
| Residuals | 48.000 | 4.353 | 0.091 |  | 0.678 |  |  |  |  |

Supplementary table S4: PCA from reduced set of Bioclim variables.

| **PCA** | PC1 | PC2 | PC3 | PC4 | PC5 |
| --- | --- | --- | --- | --- | --- |
| Standard deviations: | 1.931 | 1.544 | 0.742 | 0.568 | 0.112 |
| Rotation: | PC1 | PC2 | PC3 | PC4 | PC5 |
| BIO4 | -0.175 | 0.452 | 0.832 | -0.234 | 0.008 |
| BIO1 | -0.470 | -0.251 | -0.006 | -0.289 | 0.002 |
| BIO17 | -0.441 | 0.134 | 0.076 | 0.843 | -0.253 |
| BIO16 | -0.108 | 0.587 | -0.427 | -0.296 | -0.608 |
| BIO12 | -0.313 | 0.488 | -0.326 | 0.015 | 0.743 |
| BIO11 | -0.457 | -0.295 | -0.109 | -0.150 | -0.002 |
| BIO10 | -0.486 | -0.210 | 0.045 | -0.204 | -0.116 |
| Importance of components | PC1 | PC2 | PC3 | PC4 | PC5 |
| Standard deviation | 1.931 | 1.544 | 0.742 | 0.568 | 0.112 |
| Prop. of variance | 0.533 | 0.341 | 0.079 | 0.046 | 0.002 |
| Cumulative prop. | 0.533 | 0.873 | 0.952 | 0.998 | 1.000 |

Permutational Multivariate Analysis of Variance Using Distance Matrices

Df SumsOfSqs MeanSqs F.Model R2 Pr(>F)

Species 5 40.668 8.1337 20.343 0.82887 0.001

Residuals 21 8.396 0.3998 0.17113

Total 26 49.065 1.00000

Supplemenary Table S5: jModeltest scores for each locus. Top 8 models from each analysis shown. 24 models considered to facilitate utilization in BEAST. Selected models are in bold and outlined.

| Model | -lnL | AICc | deltaAICc | Weight | cumWeight |
| --- | --- | --- | --- | --- | --- |
| **ND2** |  |  |  |  |  |
| **GTR+I** | **3738.2668** | **7665.0336** | **0** | **0.9835** | **0.9835** |
| GTR+G | 3742.5781 | 7673.6562 | 8.6226 | 0.0132 | 0.9967 |
| GTR+I+G | 3742.8446 | 7676.5366 | 11.5029 | 0.0031 | 0.9998 |
| HKY+I | 3751.789 | 7682.7326 | 17.699 | 1.00E-04 | 0.9999 |
| HKY+G | 3752.9569 | 7685.0686 | 20.0349 | 0 | 1 |
| HKY+I+G | 3752.664 | 7686.8125 | 21.7788 | 0 | 1 |
| GTR | 3822.6546 | 7831.4662 | 166.4325 | 0 | 1 |
| HKY | 3828.7779 | 7834.3852 | 169.3516 | 0 | 1 |
| **POMC** |  |  |  |  |  |
| **HKY+I** | **2360.8772** | **4899.4622** | **0** | **0.4328** | **0.4328** |
| GTR+I | 2356.4666 | 4899.833 | 0.3707 | 0.3596 | 0.7924 |
| HKY+I+G | 2361.0545 | 4902.1089 | 2.6467 | 0.1152 | 0.9076 |
| GTR+I+G | 2356.6992 | 4902.6058 | 3.1436 | 0.0899 | 0.9975 |
| HKY+G | 2366.5045 | 4910.7168 | 11.2546 | 0.0016 | 0.9991 |
| GTR+G | 2362.4418 | 4911.7833 | 12.3211 | 9.00E-04 | 1 |
| SYM+I | 2373.4536 | 4926.9072 | 27.445 | 0 | 1 |
| K80+I | 2379.4004 | 4929.6551 | 30.1929 | 0 | 1 |
| SYM+I+G | 2376.6717 | 4935.6395 | 36.1773 | 0 | 1 |
| **C-MYC** |  |  |  |  |  |
| SYM+G | 2477.1653 | 5133.3746 | 0 | 0.5248 | 0.5248 |
| **GTR+G** | **2474.8581** | **5135.586** | **2.2114** | **0.1737** | **0.6985** |
| SYM+I+G | 2477.1692 | 5135.6542 | 2.2795 | 0.1679 | 0.8664 |
| GTR+I+G | 2474.8618 | 5137.8756 | 4.501 | 0.0553 | 0.9217 |
| K80+G | 2484.4432 | 5138.8787 | 5.5041 | 0.0335 | 0.9552 |
| HKY+G | 2481.314 | 5139.404 | 6.0293 | 0.0257 | 0.9809 |
| K80+I+G | 2484.447 | 5141.144 | 7.7694 | 0.0108 | 0.9917 |
| HKY+I+G | 2481.3178 | 5141.6798 | 8.3051 | 0.0083 | 1 |
| **RAG1** |  |  |  |  |  |
| **HKY+I** | **2360.8772** | **4899.4622** | **0** | **0.4328** | **0.4328** |
| GTR+I | 2356.4666 | 4899.833 | 0.3707 | 0.3596 | 0.7924 |
| HKY+I+G | 2361.0545 | 4902.1089 | 2.6467 | 0.1152 | 0.9076 |
| GTR+I+G | 2356.6992 | 4902.6058 | 3.1436 | 0.0899 | 0.9975 |
| HKY+G | 2366.5045 | 4910.7168 | 11.2546 | 0.0016 | 0.9991 |
| GTR+G | 2362.4418 | 4911.7833 | 12.3211 | 9.00E-04 | 1 |
| SYM+I | 2373.4536 | 4926.9072 | 27.445 | 0 | 1 |
| K80+I | 2379.4004 | 4929.6551 | 30.1929 | 0 | 1 |
